# Supplementary material for: Characterization of Volatile Compounds by HS-GC-IMS and Chemical Composition Analysis of Colored Highland Barley Roasted at Different Temperatures
Source: Foods. 2022 Sep 19;11(18):2921. doi: 10.3390/foods11182921 (PMC9498828; doi:10.3390/foods11182921)
Supplement: Supplementary file 1 [file foods-11-02921-s001.zip › foods-1892272-supplementary.pdf]

Supplemental Materials

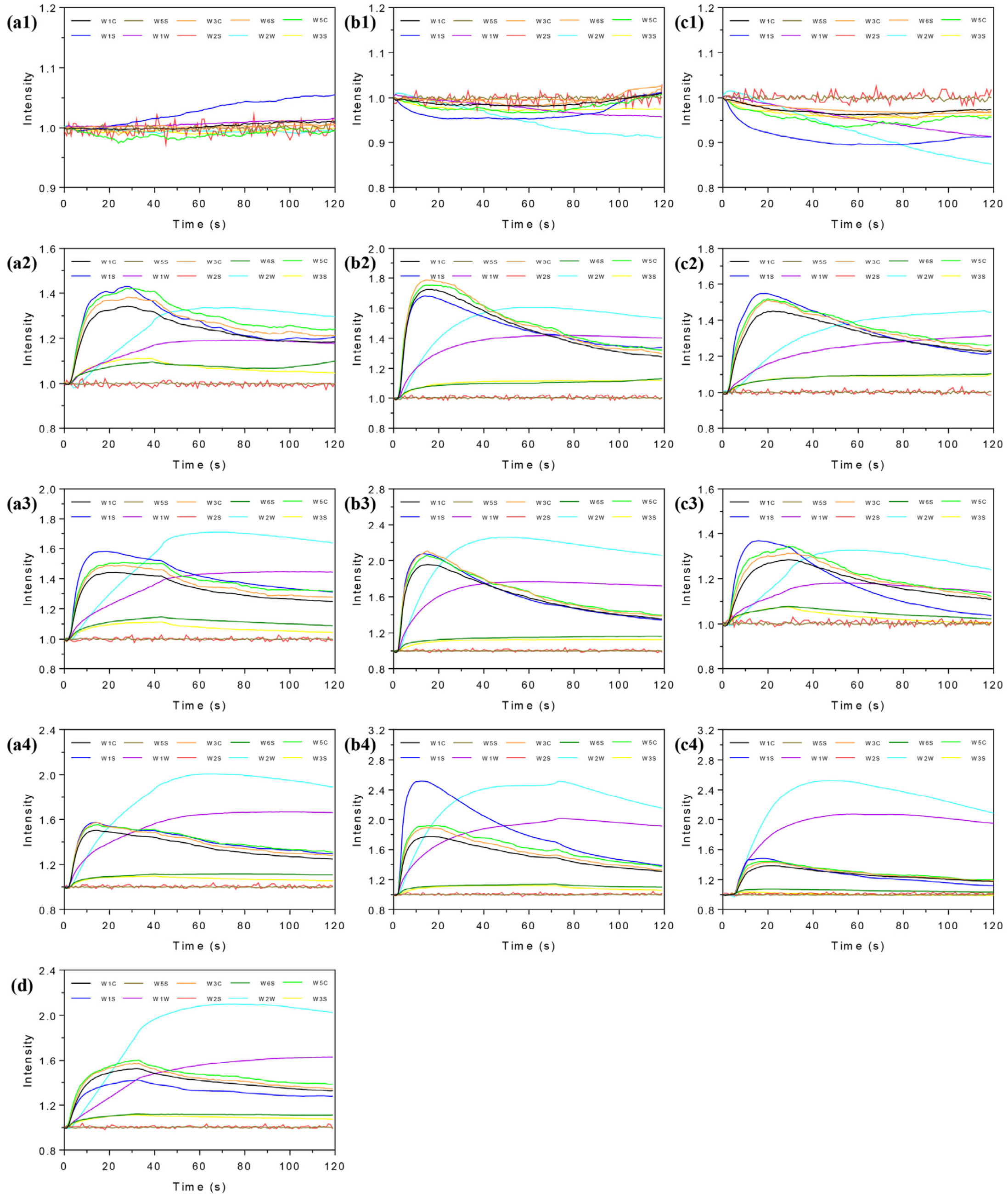

F

Figure S1 Ten sensors' responses to the aromas attributed by the E-nose in the raw and CHB roasted at different temperatures. a (white): a1 (0.0 °C); a2 (180.0 °C); a3 (220.0 °C); a4 (260.0 °C). b (blue):

b1 (0.0 ℃); b2 (180.0 ℃); b3 (220.0 ℃); b4 (260.0 ℃). c (black): c1 (0.0 ℃); c2 (180.0 ℃); c3 (220.0 ℃); c4 (260.0 ℃). d: commercial roasted barley.

a

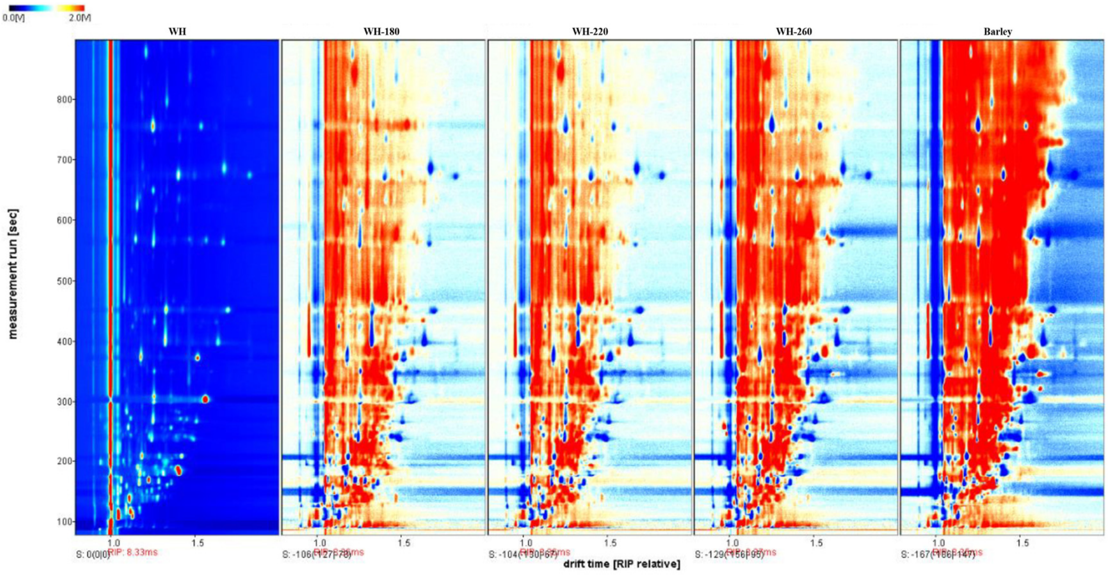

0.0[V]
2.0[V]
WH
WH-180
WH-220
WH-260
Barley
measurement run [sec]
drift time [RIP relative]
RIP: 8.33ms

b

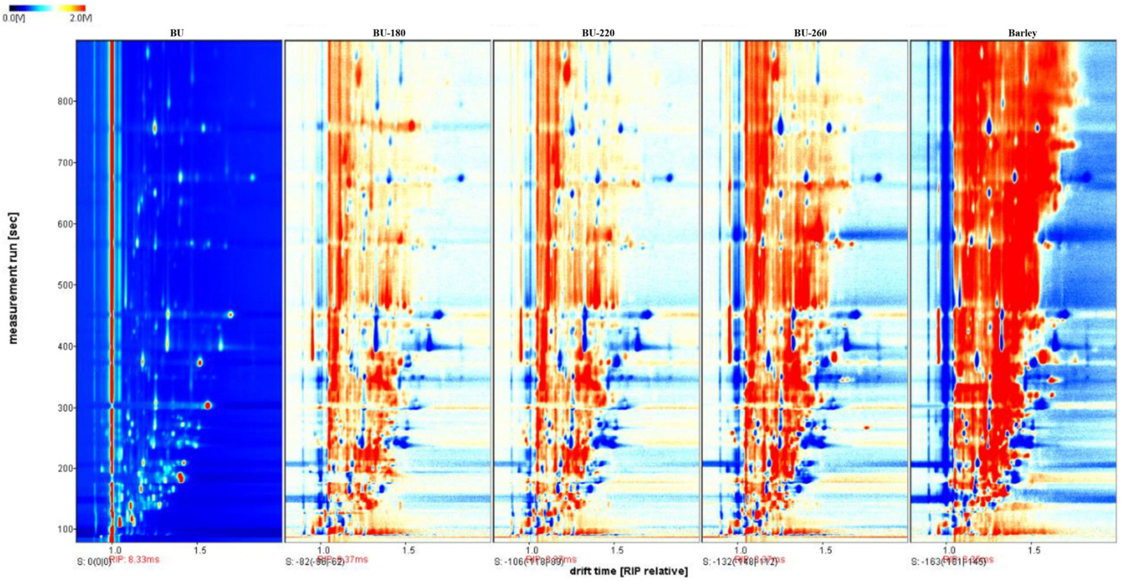

0.0[V]
2.0[V]
BU
BU-180
BU-220
BU-260
Barley
measurement run [sec]
drift time [RIP relative]
RIP: 8.33ms

c

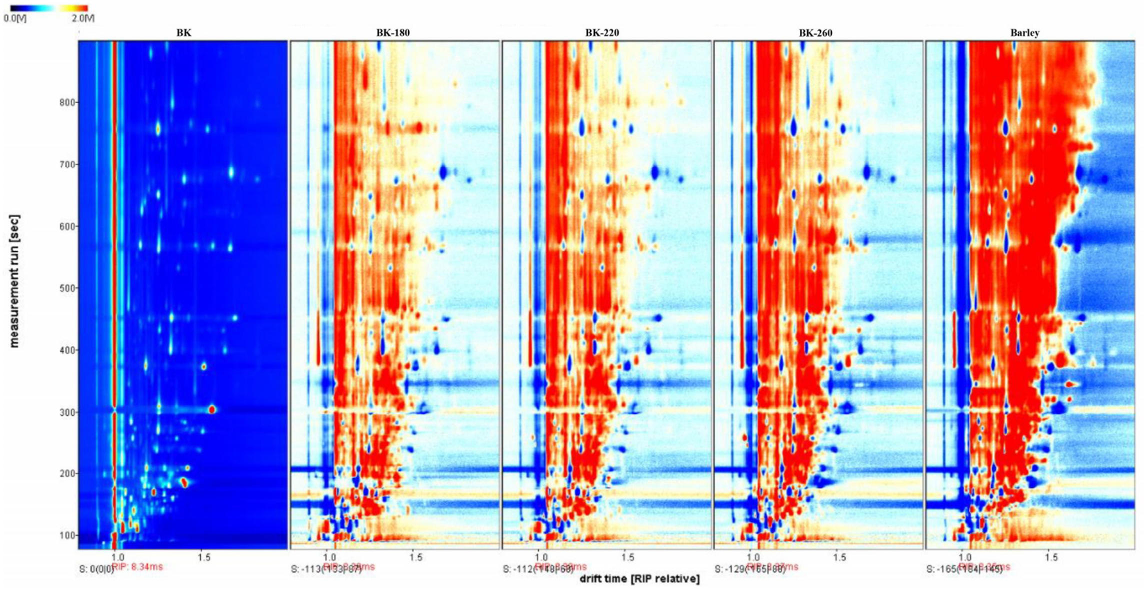

Figure S2 HS-GC-IMS topographic plots for the raw and CHB roasted at different temperatures. a: white cultivar; b: blue cultivar; c: black cultivar. Commercial roasted barley was set as the control.

Tables

Table S1 The main applications of sensors in PEN3.

Numbers	Sensor name	Substances for sensing	Reference
1	W1C	Aromatic compounds	Toluene, 10 ppm
2	W5S	Nitrogen oxides	NO_2, 1 ppm
3	W3C	Ammonia, Aromatic compounds	Benzene, 10 ppm
4	W6S	Hydrogen	H_2, 100 ppb
5	W5C	Alkanes, Less polar compounds	Propane, 1 ppm
6	W1S	Sensitive to methane (environment) *ca*. 10 ppm, Broad range, similar to No.8	CH_4, 100 ppm
7	W1W	Sulfur compounds, H2S 0.1 ppm, Terpenes, Sulfur organic compounds	H_2S, 1 ppm
8	W2S	Alcohols, Partially aromatic compounds, Broad range	CO, 100 ppm
9	W2W	Aromatic compounds, Sulfur organic compounds	H_2S, 1 ppm
10	W3S	Methane at high concentrations > 100 ppm, Sometimes very selective	CH_4, 10CH_4, 100 ppm
